# N-phenylmaleimide induces bioenergetic switch and suppresses tumor growth in glioblastoma tumorspheres by inhibiting SLC25A11

**DOI:** 10.1186/s12935-025-03813-y

**Published:** 2025-05-22

**Authors:** Hye Joung Cho, Jihwan Yoo, Ran Joo Choi, Jae-Seon Lee, Ryong Nam Kim, Junseong Park, Ju Hyung Moon, Eui Hyun Kim, Wan-Yee Teo, Jong Hee Chang, Soo-Youl Kim, Seok-Gu Kang

**Affiliations:** 1 Department of Neurosurgery, Brain Tumor Center, Severance Hospital, Yonsei University College of Medicine, Seoul, Republic of Korea; 2 Brain Tumor Translational Research Laboratory, Department of Biomedical Sciences, Yonsei University College of Medicine, Seoul, Republic of Korea; 3 Department of Neurosurgery, Gangnam Severance Hospital, Yonsei University College of Medicine, Seoul, Republic of Korea; 4 Department of Medical Science, Yonsei University Graduate School, Seoul, Republic of Korea; 5 Department of Division of Cancer Biology, National Cancer Center, Goyang, Republic of Korea; 6 Department of College of Medicine, The Catholic University of Korea, Catholic Medical Center, Seoul, Republic of Korea; 7 Cancer and Stem Cell Biology Program, Duke-NUS Medical School, Singapore, Singapore; 8 Institute of Molecular and Cell Biology, A*STAR, Singapore, Singapore; 9 Brain Research Institute, Yonsei University College of Medicine, Seoul, 03722 Republic of Korea

**Keywords:** Bioenergetics, Glioblastoma, KN612, Malate-aspartate shuttle, SLC25A11

## Abstract

**Background:**

Glioblastoma (GBM) is a highly resistant tumor, and targeting its bioenergetics could be a potential treatment strategy. GBM cells depend on cytosolic nicotinamide adenine dinucleotide (NADH), which is transported into the mitochondria via the malate-aspartate shuttle (MAS) for ATP production. N-phenylmaleimide (KN612) is a MAS inhibitor that targets SLC25A11, an antiporter protein of the MAS. Therefore, this study investigated the effects of KN612 in GBM treatment using in vitro and in vivo models.

**Methods:**

We examined the biological effects of KN612 in GBM tumorspheres (TSs), including its effects on cell viability, ATP level, cell cycle, stemness, invasive properties, energy metabolic pathways, and transcriptomes. Additionally, we investigated the in vivo efficacy of KN612 in a mouse orthotopic xenograft model.

**Results:**

Transcriptomic analysis showed that SLC25A11 mRNA expression was significantly higher in GBM TSs than in normal human astrocytes. Additionally, siRNA-mediated SLC25A11 knockdown and KN612-mediated MAS inhibition decreased the oxygen consumption rate, ATP levels, mitochondrial activity, and cell viability in GBM TSs and decreased the stemness and invasion ability of GBM cells. Moreover, gene ontology functional annotation indicated that KN612 treatment inhibited cell-cycle and mitotic processes. Furthermore, KN612 treatment reduced tumor size and prolonged survival in an orthotopic xenograft model.

**Conclusions:**

Targeting GBM bioenergetics using KN612 may represent a novel and effective approach for GBM treatment.

**Supplementary Information:**

The online version contains supplementary material available at 10.1186/s12935-025-03813-y.

## Background

Glioblastoma (GBM) represents the most common malignant primary brain tumor [1–4], with poor diagnosis despite available treatments, including neurosurgery, radiotherapy, and chemotherapy. Although studies have reported the clinical benefits of bevacizumab, supratotal resection, and antiepileptic drugs, the overall survival of patients is approximately 15–20 months [3, 5, 6]. Additionally, recent clinical trials with immunotherapy, including immune checkpoint inhibitors, showed disappointing results without a survival benefit [7–9]. Therefore, unconventional approaches are required to effectively treat GBM, and targeting bioenergetics could be a novel option.

Generally, malignant cells utilize five to ten times more glucose during glycolysis and produce more lactate than normal cells in the presence of oxygen, a phenomenon known as the Warburg effect [10]. Notably, the high glycolytic rate of malignant cells results in a high cytosolic NADH/NAD^+^ ratio; therefore, it is crucial for the NADH shuttle, including the malate-aspartate shuttle (MAS) that transports cytosolic NADH into the mitochondria, to maintain the NADH equilibrium to ensure the continuation of glycolysis [11]. Importantly, a previous study identified high concentrations of a NADH isoform in GBM [2]. Moreover, inhibiting aldehyde dehydrogenase (ALDH), a key enzyme in NADH production, along with oxidative phosphorylation (OxPhos), reduces ATP levels, stemness, invasiveness, and cell viability in GBM tumorspheres (GBM TSs). Furthermore, ALDH and OxPhos inhibition exhibited a synergistic effect with the GBM chemotherapeutic drug temozolomide, effectively interfering with energy production and inhibiting cell proliferation in GBM TSs [12].

Among these energy systems, the MAS plays a major role in moving cytosolic NADH into the mitochondria to produce ATP [13]. Notably, the MAS consists of four different enzymes, malate dehydrogenase (MDH), glutamic-oxaloacetic transaminase (GOT), and two antiporters, mitochondrial aspartate-glutamate carrier 1 (AGC1) and oxaloglutarate carrier (OGC, SLC25A11) [13, 14]. Aminooxyacetic acid-induced MAS inhibition decreased ATP production and increased apoptosis or necrosis in C6 glioma cell line [11]. Additionally, SLC25A11 knockdown inhibited cancer cell proliferation by reducing ATP production by 80% and induced cell cycle arrest in lung and melanoma cancer cells [13]. Although N-phenylamide (KN612), a SLC25A11 inhibitor, has been shown to interfere with cancer growth by reducing ATP production in several studies [15], its effect and mechanism in GBM remain unclear.

Therefore, we aimed to investigate the effect of KN612 on GBM TSs as a potential regulator of bioenergetic flux in GBM. Specifically, we investigated the effect of KN612 on SLC25A11 expression and MAS-mediated mitochondrial energy production in GBM TSs. Additionally, we examined the effects of KN612 on mitochondrial activity and GBM cell viability and assessed its therapeutic potential in an in vivo orthotopic xenograft model.

## Methods

### Cell culture and reagents

Briefly, we generated three primary tumor cell lines (TS13-30, TS13-64 and TS15-88 cells) from tissues obtained from patients with GBM (Supplementary Table 1). Additionally, normal human astrocytes (NHA) were purchased from Lonza and used as controls in this study. Three TS-forming GBM cells were obtained from GBM tissue specimens with approval from the Institutional Review Board of Yonsei University College of Medicine (4-2021-1319). TS was isolated from human GBM tumor tissues as previously described [2, 12]. For TS culture, cells were cultured in TS complete medium composed of Dulbecco’s modified Eagle’s medium/F12 (Mediatech, Manassas, VA, USA), 1 × B27 (Invitrogen, San Diego, CA, USA), 20 ng/mL of basic fibroblast growth factor, and 20 ng/mL of epidermal growth factor (Sigma-Aldrich, ST. Louis, MO, USA). NHA cells were obtained commercially (Lonza, Walkersville, MD, USA) and cultured in Astrocyte Basal Medium supplemented with the Astrocyte Growth Kit (Lonza). Notably, the NHA culture protocol was adapted from previously reported studies and followed the manufacturer’s instructions [16]. Cells were maintained at 37 °C in a humidified incubator with 5% CO₂, and the medium was replaced every 3–4 days. All in vitro experiments were performed under TS conditions, and KN612 (Sigma-Aldrich) was dissolved in dimethyl sulfoxide (DMSO).

### Transcriptome data analysis and processing

Total RNA was extracted from GBM tissues and TS and sequenced, followed by quality assessment of raw reads and removal of adapters using fastQC (v.0.10.1) and Trimmomatic (v. 0.38), respectively. Low-quality reads were filtered based on the following criteria: reads with more than 10% 'N's, over 40% bases with quality scores below 20, and average quality scores under 20. High-quality reads were aligned to the human reference genome using Tophat27. Gene expression levels were quantified using Cufflinks v2.1.128 with the Ensembl gene annotation database (release 72). Pearson’s correlation analysis was conducted using the GENE-E software, and expression levels were visualized as heatmaps.

### Transfection with small interfering RNAs (siRNAs)

To induce SLC25A11 knockdown, cells were transfected with an siRNA: si-*SLC25A11*#1 (5′-3′; GGAAUACAAGAACGGGCUGGACGUGdTdT). Cells transfected with the scrambled siRNA were used as a nonspecific control. Specifically, GBM TSs were transfected with the siRNA duplexes for 48 h in Opti-MEM (Invitrogen) using Lipofectamine 3000 (Invitrogen). Western blotting was performed to confirm the silencing efficiency.

### Assessment of ATP production and cell viability

Briefly, GBM TSs were plated in 96-well plates (5 × 10^3^ cells per well) and treated with KN612 for 72 h after a 24 h incubation period. ATP levels and cell viability were assessed using the CellTiter-Glo assay kit (Promega, Madison, MI, USA) and MTT assay, respectively. For the MTT assay, 10 µL of MTT solution (5 mg/mL in PBS) was added to each well, followed by incubation at 37 °C for 1 h. Formazan crystals were dissolved with 100 µL of DMSO, and absorbance was recorded at 570 nm. ATP levels were assessed by adding the CellTiter-Glo reagent and measuring luminescence. Each experiment was repeated three times in triplicate, and results were expressed as the percentage of viable cells relative to the controls.

### XF cell mito stress analysis

Briefly, the oxygen consumption rate (OCR) of GBM TSs treated with KN612 for 24 h was measured using a Seahorse XFe96 instrument (Agilent, Seahorse Bioscience, North Billerica, MA, USA). After incubating in base medium for 1 h, we measured OCR following sequential addition of 1 μM oligomycin, 0.25 μM of trifluoromethoxy carbonylcyanide phenylhydrazone (FCCP), and 0.5 μM of rotenone/antimycin A. OCR data were normalized to cellular protein content determined using a BSA assay.

### Measurement of mitochondrial membrane potential (∆ψm)

GBM TSs were plated in 60 mm culture dishes and treated with KN612 for 72 h. Cells in the positive control group were treated with 20 μM of FCCP (C2920, Sigma-Aldrich) for 30 min. Thereafter, the cells were treated with 50 nM of tetramethylrhodamine (TMRE) for 20 min at 37 °C, followed by washing and resuspension in PBS/BSA. Finally, the mitochondrial membrane potential was analyzed at Ex/Em = 549/575 nm using using a flow cytometer (BD Biosciences, Franklin Lakes, NJ, USA).

### Liquid chromatography-mass spectrometry measurement (LCMS-MS)

GBM TSs treated with KN612 for 48 h were subjected to metabolite extraction using methanol/water, followed by liquid–liquid extraction with chloroform. LC–MS/MS analysis was performed to analyze energy metabolism-related metabolites. Multiple reaction monitoring in negative ion mode was utilized for quantification, with normalization to an internal standard. Metabolite levels were further normalized to protein content.

### JC-1 assay

Briefly, the Mitochondrial membrane potential (ΔΨm) of KN612-treated GBM TSs was evaluated using a JC-1 mitochondrial membrane potential detection kit (Cell Signaling Technology, Beverly, MA, USA). The kit included the cationic dye, JC-1 (5,5′,6,6′-tetrachloro-1,1′,3,3′-tetraethyl-imidacarbocyanine iodide) and the mitochondrial membrane potential disruptor 2-[2-(3-chlorophenyl) hydrazinylidene]-propanedinitrile (CCCP). Specifically, cells were incubated with 40 nM of JC-1 at 37 °C for 15 min, followed by flow cytometry (BD Biosciences). Green and red fluorescence intensities were measured at 514/529 nm (FL-1) and 585/590 nm (FL-2), respectively. Control experiments involved treatment with 50 μM of CCCP to disrupt ΔΨm.

### Annexin-V assay

Cell apoptosis was assessed using the FITC Annexin V Apoptosis Detection Kit I (Sigma-Aldrich). Briefly, cells at a concentration of 1 × 10^6^ cells/mL were suspended in binding buffer, stained with FITC-annexin V and propidium iodide, and incubated at 4 °C for 15 min. Thereafter, the samples were analyzed using flow cytometer (Beckman Coulter, Brea, CA, USA).

### Neurosphere formation assay

Briefly, 10 single GBM TSs were cultured in 96-well plates and divided into control and drug-treated groups. After 2 weeks, sphere-positive wells were counted, and the proportion relative to the control was calculated. Images were analyzed using ToupView software (ToupTek Photonics, Hangzhou, Zhejiang, China).

### 3D invasion

Single GBM TSs were seeded in individual wells of a 96-well plate filled with a mixed matrix of Matrigel, type I collagen (Corning, Corning, NY, USA), and TS complete medium. Spheroids were seeded before gelation, and complete medium was added to prevent drying. The invaded area was quantified as the area occupied (72 h − 0 h)/0 h.

### Western blot analysis

Briefly, protein extracts were separated using sodium dodecyl sulfate polyacrylamide gel electrophoresis on 10% Tris–glycine gels and transferred onto nitrocellulose membranes. After blocking with a blocking buffer, the membranes were incubated with specific primary antibodies overnight at 4 °C. The primary antibodies were Sox2 (Merck Millipore, Darmstadt, Germany 1:1000), MDH1, MDH2, and Nestin (Novus Biologicals, Littleton, CO, USA, 1:1000), CD133, PDPN, Zeb1, and Snail (Cell Signaling Technology, MA, USA, 1:1000), N-cadherin (R&D Systems, Minneapolis, MN, USA), SLC25A11, Oct3/4, Twist, and GAPDH (Santa Cruz Biotechnology, Santa Cruz, CA, USA, 1:2000). Details of the antibodies used in the study were listed in supplementary Table 2. Images were acquired using an ImageQuant LAS 4000 Mini System (GE Healthcare Life Sciences, Little Chalfont, UK). Western blot was performed in triplicate to ensure reproducibility of the results.

### Gene ontology enrichment and REACTOME pathway analyses

Gene ontology enrichment analysis was performed using the “clusterProfiler” and “DOSE” packages in R. MAS pathway analysis was executed using the “ReactomePA” package in R.

### Mouse orthotopic tumor models

All animal experiments were approved by the Institutional Animal Care and Use Committee of Yonsei University College of Medicine. Male nude mice (8-week-old) were provided by the Central Lab Animal. TS13-64-luc TSs were pre-treated with KN612 (10 μ M) for 72 h. TS13-64-luc TSs in the control (n = 5) and KN612 (n = 5) groups were implanted into the right frontal lobe of the mice using a guide-screw system [4]. Live cells were counted using trypan blue staining, and each mouse was injected with 5 × 10^5^ cells to a depth of 4.5 mm. At the end of the experimental period, tumor tissues were sampled and cut into 4 μm thick sections for immunohistochemistry using a microtome. SLC25A11 and Zeb1 were detected using the peroxidase/3,3'-diaminobenzidine staining system [17]. To quantify Zeb1-positive cells, we defined invasive cells as Zeb1-positive cells located beyond the tumor boundary, identified by hematoxylin and eosin (H&E) staining. Ten images were randomly captured around the tumor periphery were captured at × 40 magnification, and Zeb1-positive cells were analyzed using a semi-quantitative scoring system [17, 18].

### Bioluminescence imaging

Bioluminescence imaging and analysis were performed using an IVIS imaging system and Living Image v4.2 software (Caliper Life Sciences, Hopkinton, MA, USA). Mice were anesthetized with 2.5% isoflurane 15 min prior to signal acquisition, and 100 μL of D-luciferin (30 mg/mL dissolved in PBS, 100 μL; Promega) was intraperitoneally injected.

### Statistical analysis

All graph generation and statistical analyses were conducted using GraphPad Prism 9 (GraphPad Software Inc., San Diego, CA, USA), and R software (version 4.3.3). Significant differences among treatment groups with unequal variance were assessed using Welch’s ANOVA and the Games-Howell post hoc tests, performed in R software. Comparisons between two independent groups were performed Student’s t-test. Survival data were analyzed using the Kaplan–Meier method and compared using log-rank tests. Statistical significance was set at *p < 0.05, **p < 0.01, ***p < 0.001.

## Results

### SLC25A11 knockdown affects energy metabolism in GBM TSs

**Fig. 1:**
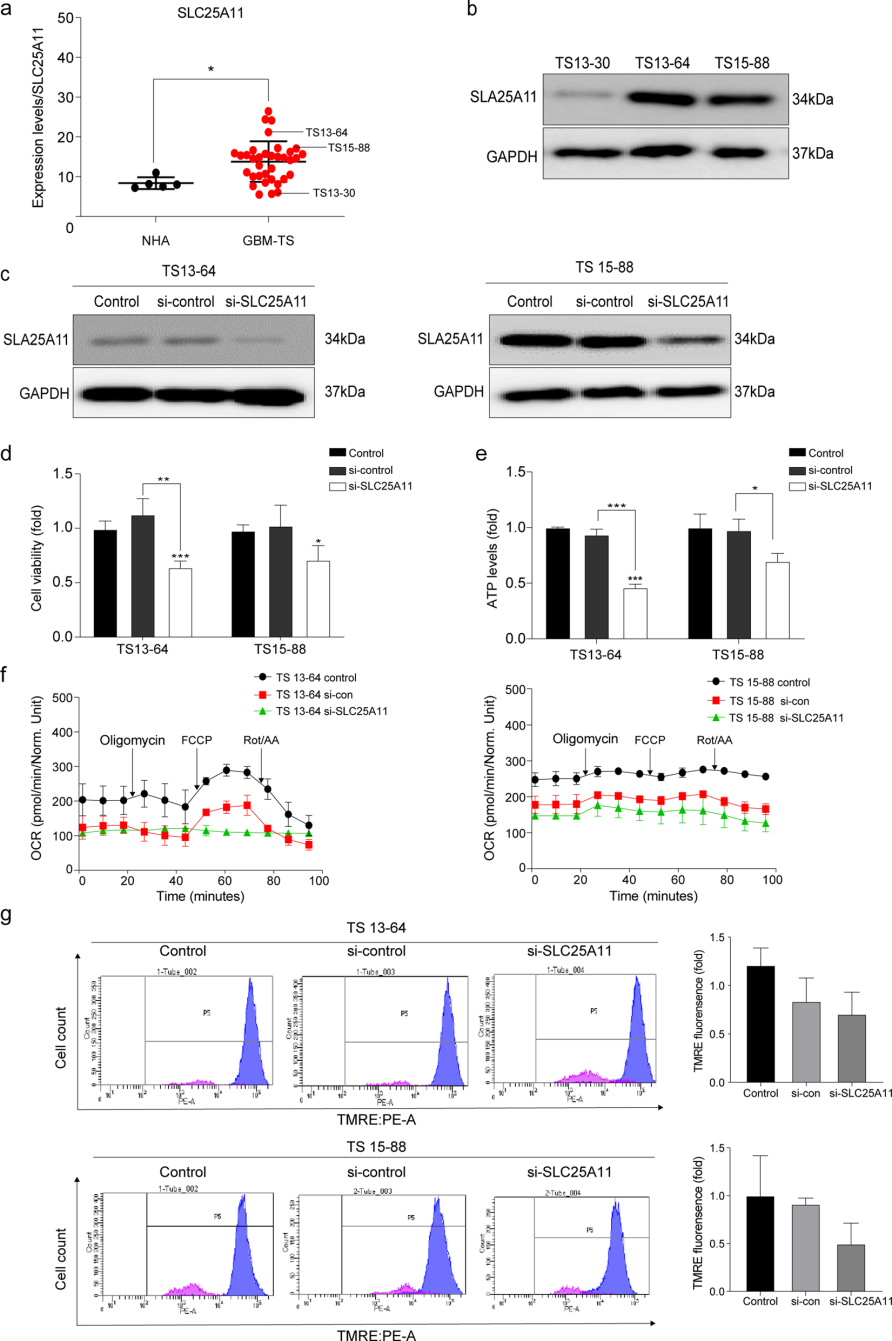
Targeting the SLC25A11 gene in GBM TSs. Cell viability, ATP production and mitochondrial function were measured 48 h after transfection with si-SLC25A11. **a** SLC25A11 expression levels in normal human astrocytes (NHA; n = 5) and GBM TS (n = 35) were determined using RNA-Seq. **b** Western blotting to detect SLC25A11 expression in GBM TSs. **c** Western blotting to detect SLC25A11 expression in TS13-64 and TS15-88 following transfection with siRNA. **d** Cell viability and **e** ATP levels were measured in TS13-64 and TS15-88 after SLC25A11 knockdown. **f** Oxygen consumption rate (OCR) was measured in TS13-64 and TS15-88 after SLC25A11 knockdown. **g** Mitochondrial membrane potential was measured in TS13-64 and TS15-88 after SLC25A11 knockdown. Differences between groups were evaluated using Welch’s ANOVA and Games-Howell post hoc tests. The data are presented as the mean ± standard deviation (SD); *p < 0.05, **p < 0.01 and ***p < 0.001

Transcriptome analysis confirmed that SLC25A11 mRNA expression levels were significantly higher in GBM TSs than in normal human astrocytes (Fig. 1a). To investigate the role of SLC25A11 in GBM, we examined protein expression in three GBM TSs. Notably, the results were consistent with the transcriptome data, confirming high SLC25A11 expression in TS13-64 and TS15-88 (Fig. 1b; Supplementary fig. S2a). To further explore the role of SLC25A11 in GBM, we evaluated the impact of siRNA-mediated SLC25A11 knockdown on cell viability and ATP levels in GBM TSs. Successful SLC25A11 knockdown was confirmed using western blot analysis. Transfection with si-*SLC25A11* significant decreased SLC25A11 protein expression in the cells (Fig. 1c; Supplementary fig. S2b). Additionally, SLC25A11 knockdown significantly reduced cell viability and ATP levels in TS13-64 and TS15-88 cells compared with those in the control group (Fig. 1d, e). Moreover, SLC25A11 knockdown markedly reduced mitochondrial membrane potential and mitochondrial respiration (Fig. 1f, g). Collectively, these findings indicate that SLC25A11 plays a key role in regulating energy metabolism in GBM TSs.

### Biological effects of KN612 on GBM TSs

**Fig. 2:**
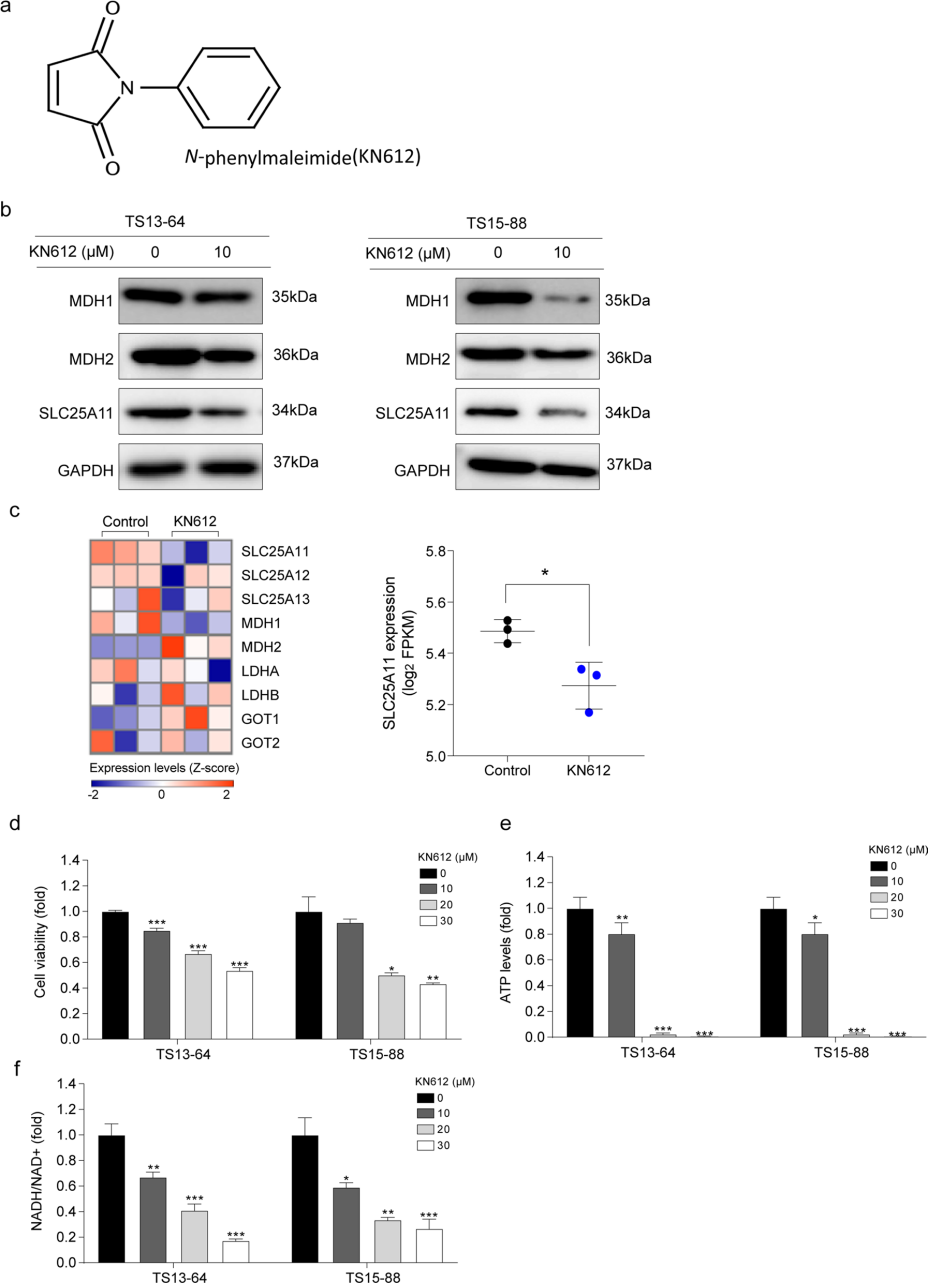
Effects of KN612 on the expression of malate-aspartate shuttle (MAS)-related genes, cell viability, and energy levels in GBM TS. **a** Chemical structure of KN612. **b** Western blotting to detect MAS-related proteins following KN612 treatment. **c** Heatmaps visualizing changes in the mRNA levels of MAS-related genes after KN612 treatment. **d** Cell viability was determined using the MTT assay. **e** ATP level was measured using the luminescent ATP assay kit. **f** NADH/NAD^+^ ratio was measured in GBMTSs treated with the indicated concentration of KN612. Differences between groups were evaluated using Welch’s ANOVA and Games-Howell post hoc tests. Data are presented as the mean ± standard deviation (SD); *p < 0.05, **p < 0.01 and ***p < 0.001

To examine the role of the energy transport system in GBM, we inhibited MAS activity using KN612 (Fig. 2a). Specifically, two GBM TSs with high SLC25A11 expression, TS13-64 and TS15-88, were treated with various concentrations of KN612 for 72 h, and the protein and mRNA expression of MAS-related factors were examined KN612 treatment significantly downregulated SLC25A11 protein and mRNA expression levels in TS13-64 and TS15-88 compared with those in the control group (Fig. 2b, c; Supplementary fig. S3a-c). Additionally, we evaluated the effects of KN612 on cell viability and ATP levels. KN612 treatment significantly decreased cell viability and ATP levels in TS13-64 and TS15-88 cells in a dose-dependent manner (Fig. 2d, e). In contrast, KN612 treatment did not significantly affect ATP production and the viability of NHA cells even at concentrations that induced cell death in GBM TSs (Supplementary fig. S1a-b). Additionally, KN612 treatment decreased cytosolic NAD + /NADH production in the cells (Fig. 2f). Overall, these findings suggest that GBM TSs rely on energy production through the MAS antiporter SLC25A11. Based on the results of the cell viability assay, a KN612 dose of 10 μM, which reduced cell viability to < 20%, was selected for subsequent experiments. Notably, this concentration was selected to avoid excessive cell death, which may hinder the accurate assessment of biological effects [19].

### Effect of KN612 on energy metabolites levels in GBM TSs

**Fig. 3:**
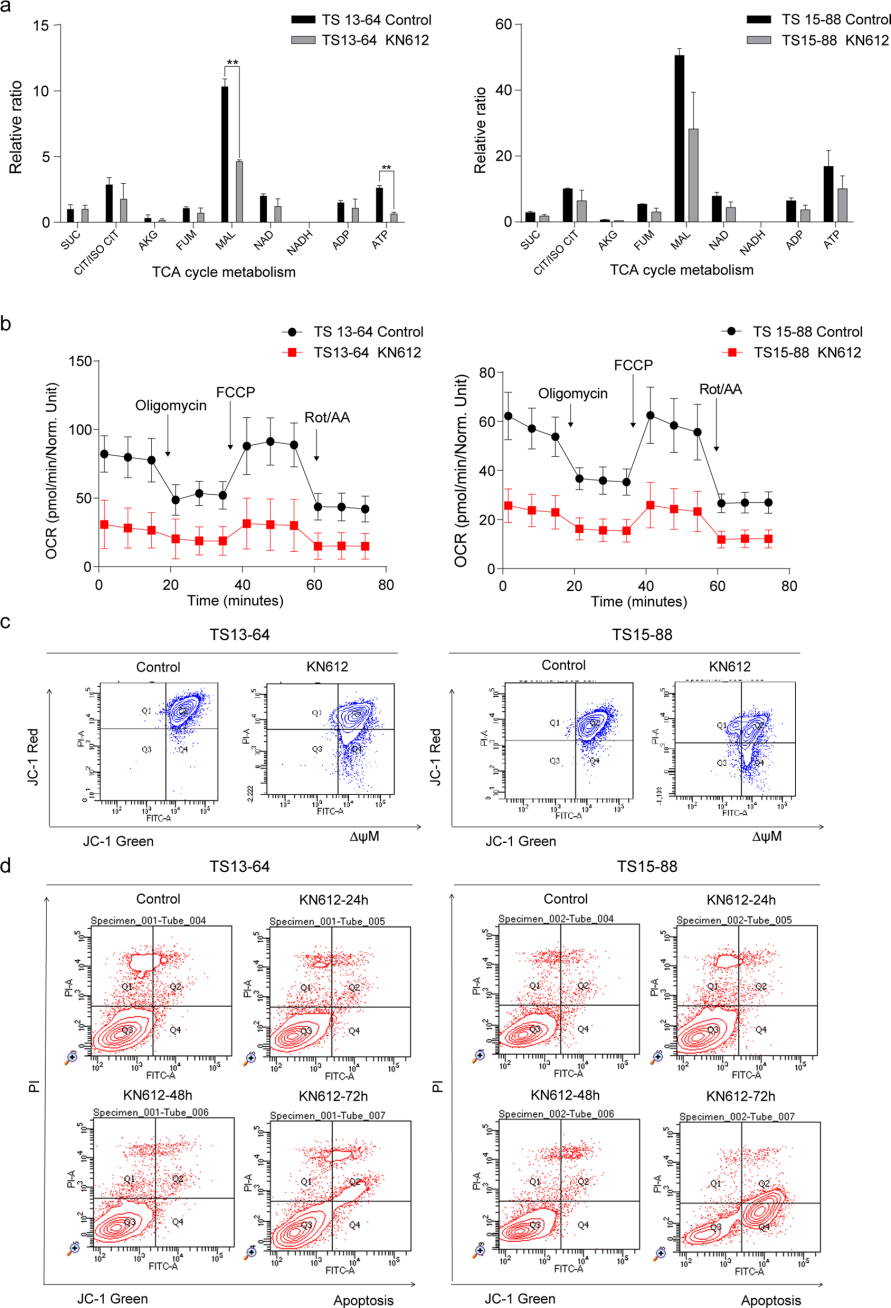
Effects of KN612 on mitochondria function and cell death in GBM TSs. Determination of mitochondrial membrane potential and energy metabolism levels was performed 72 h after KN612 treatment. **a** Metabolite levels were measured using LC–MS/MS. **b** Oxygen consumption rate (OCR) was measured using the Seahorse XFe96 analyzer. **c** Mitochondrial membrane potential (∆ψM) was assessed using JC-1 staining and flow cytometry, with green fluorescence indicating increased apoptosis. **d** Representative dot plot data illustrating both early and late apoptosis were used to assess apoptosis via the annexin V assay. *p < 0.05, **p < 0.01, and ***p < 0.001 relative to the control group

To further elucidate the effect of KN612 on cancer cell energy metabolism, we investigated energy metabolite levels in GBM TSs after 72 h treatment with KN612. KN612 treatment affected tricarboxylic acid (TCA) cycle intermediates, as evidenced by a significant decrease in malate levels compared with that in the control group. Additionally, KN612 treatment markedly decreased ATP levels and OCR in cells, indicating severe metabolic impairment (Fig. 3a, b). Furthermore, we assessed the effects of changes in energy level on mitochondrial function and apoptosis. Due to the nature of TS culture, the detection of PI-positive cell populations can also occur in the control group [20, 21]. KN612 treatment significantly decreased mitochondrial membrane potential in both TS13-64 and TS15-88 (Fig. 3c) and induced apoptosis after 72 h of treatment (Fig. 3d). Collectively, these findings indicate that KN612 effectively impairs mitochondrial function by inhibiting intracellular malate.

### Effect of KN612 on the stemness and invasiveness of GBM TSs

**Fig. 4:**
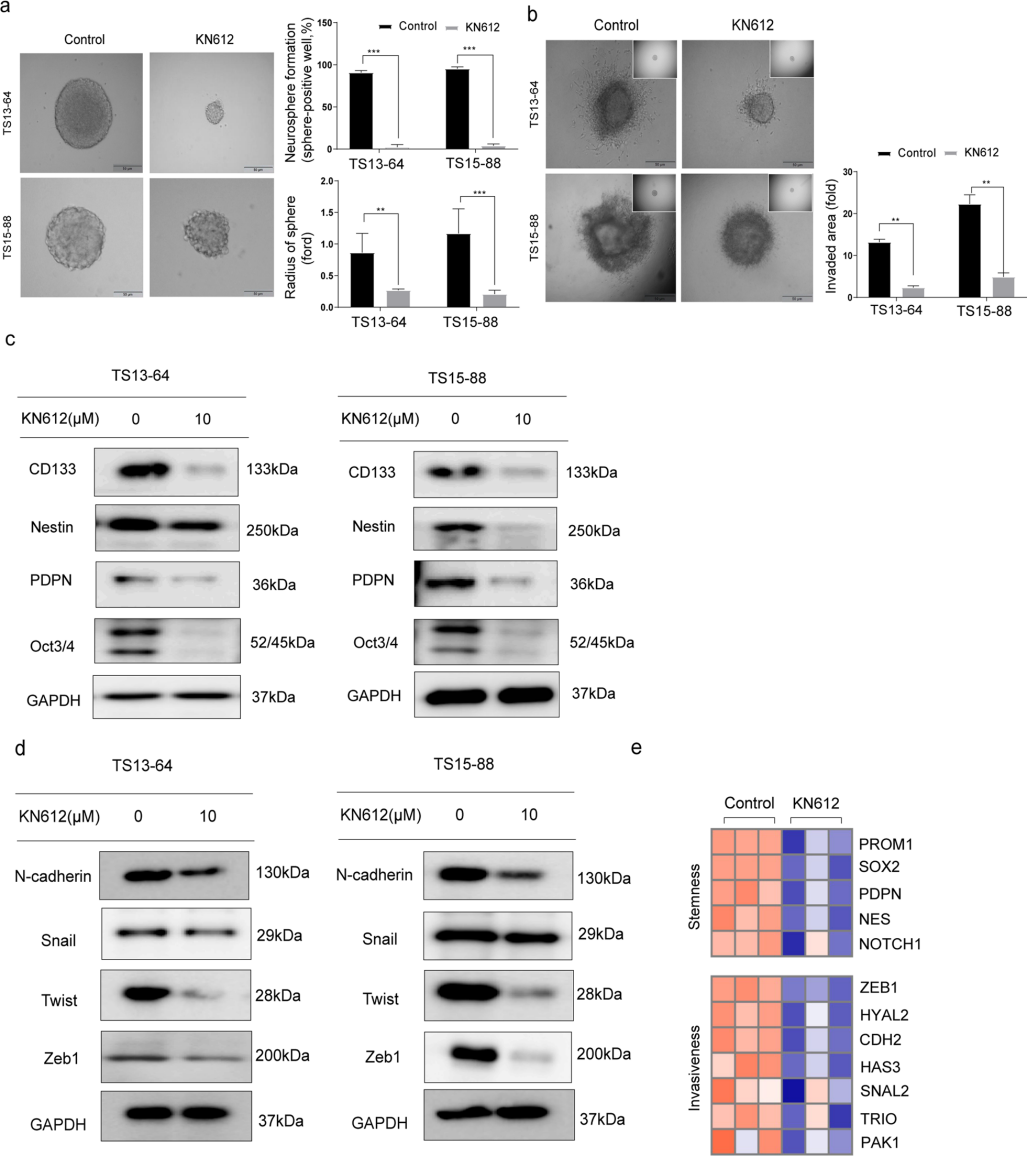
Effects of KN612 on the stemness and invasiveness of GBM TSs. Evaluation of stemness and invasiveness was performed 72 h after KN612 treatment. **a** Stemness was assessed using neurosphere formation assay, which measured both the sphere formation capacity and the percentage of sphere radius. **b** Invasive potential was assessed using a 3D invasion assay. **c** Western botting was performed to examine the expression of proteins associated with stemness and **d** invasion. **e** Heatmaps displaying expression levels of genes associated with stemness and invasion. Significant differences were determined unpaired Student's *t*-test (means ± SD; *p < 0.05, **p < 0.01, and ***p < 0.001)

In this study, we investigated the effects of KN612 on stemness and invasiveness in GBM TSs. Regarding stemness, KN612 treatment significantly decreased sphere formation and reduced sphere size in the TS13-64 and TS15-88 groups compared with those in the control group (Fig. 4a). Additionally, quantitative analysis of invaded areas revealed that KN612 treatment markedly inhibited invasiveness compared with that in the control group (Fig. 4b). Moreover, KN612 treatment significantly decreased the expression of the stemness-related proteins, including CD133, Nestin, PDPN, and OCT3/4 (Fig. 4c; Supplementary fig. S4a-c), and effectively decreased the expression of invasiveness markers such as N-cadherin, Snail, Twist, and Zeb1 (Fig. 4d; Supplementary fig. S5a-c). Consistently, RNA-seq analysis showed that KN612 treatment downregulated the mRNA expression of genes associated with stemness (*PROM1, SOX2, PDPN, NES,* and *NOTCH1*) and invasiveness (*ZEB1, HYAL2, CDH2, HAS3, SNAI2, TRIO,* and *PAK1*) compared with those in the control group (Fig. 4e). Overall, these findings suggest that KN612 suppresses stemness and invasiveness in GBM TSs, thereby effectively inhibiting the malignant progression of GBM.

### Effect of KN612 on the gene expression profile

**Fig. 5:**
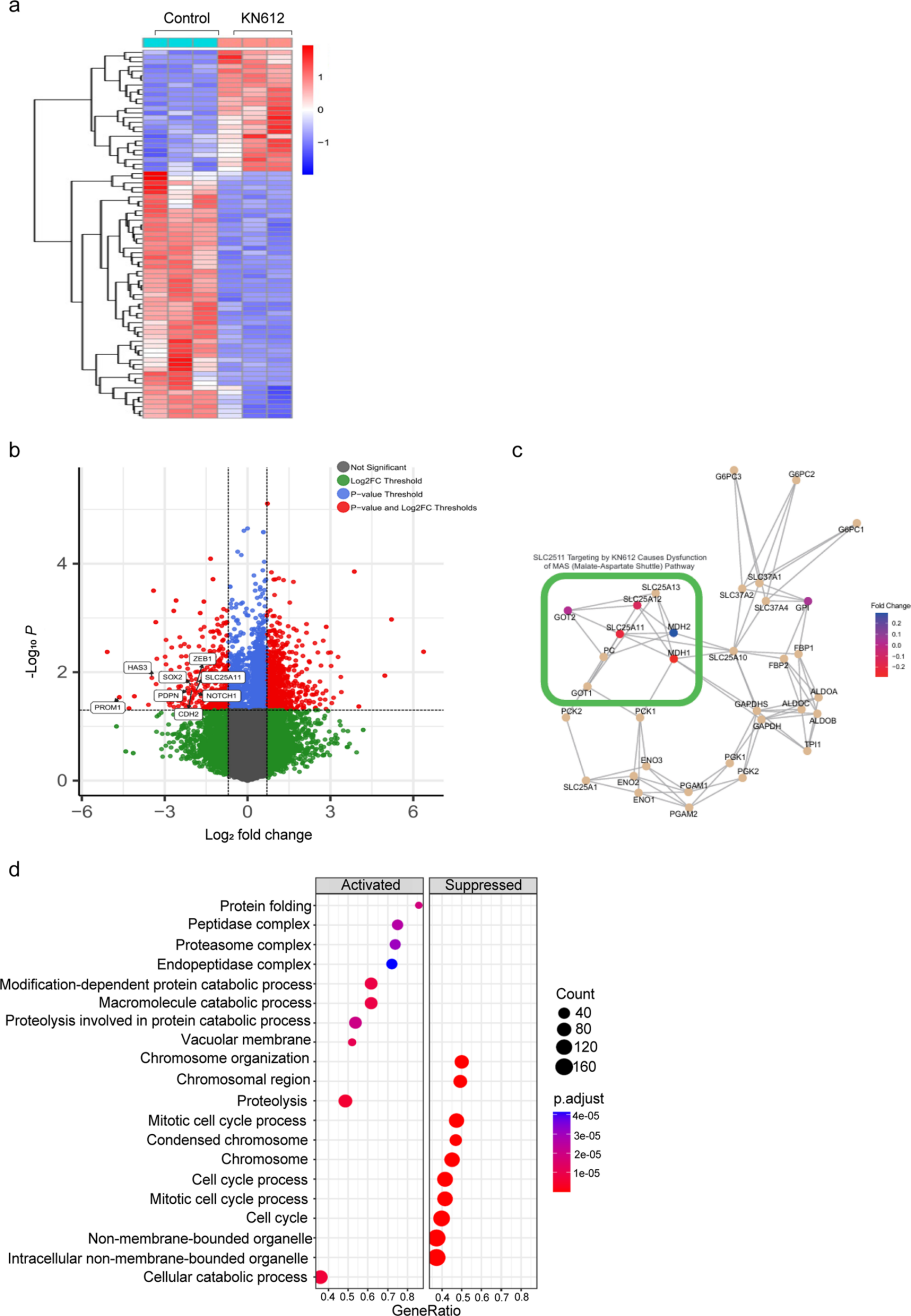
Effects of KN612 on gene expression profile. RNA-seq was performed to examine the transcriptomic profile of TS13-64 after KN612 treatment for 72 h. **a** Heatmap of DEGs between the control and KN612 treatment groups. DEGs were defined as genes with p < 0.05 and log2 fold change cutoff of 0.1. **b** Volcano plot showing DEGs based on *p*-value and fold change. Red, yellow, and blue dots indicate genes with *p* < 0.05, log2 (fold change) > 0.5, or both, respectively. **c** Visualization of genes related to the MAS pathway based on REACTOME pathway analysis. **d** Upregulated and downregulated enriched genes following KN612 treatment

RNA-seq was conducted to investigate the effect of KN612 treatment on the transcriptome profile of GBMTSs, using TS13-64. Notably, several differentially expressed genes were identified in the control vs. KN612, as indicated in a heatmap (Fig. 5a). Particularly, KN612 treatment significantly decreased the expression of genes related to the MAS antiporter SLC25A11 and stemness and invasiveness genes *ZEB1, HAS3, SOX2, PDPN, PROM1,* and *CDH2* (Fig. 5b), which was confirmed by REACTOME analysis (Fig. 5c). Moreover, gene ontology analysis showed that KN612 significantly reduced cell cycle and mitotic cell cycle processes (Fig. 5d). Collectively, these findings suggest that KN612 treatment may disrupt cell cycle progression and mitotic processes, potentially contributing to the inhibition of GBM growth and progression.

### Therapeutic responses in an orthotopic xenograft model

**Fig. 6:**
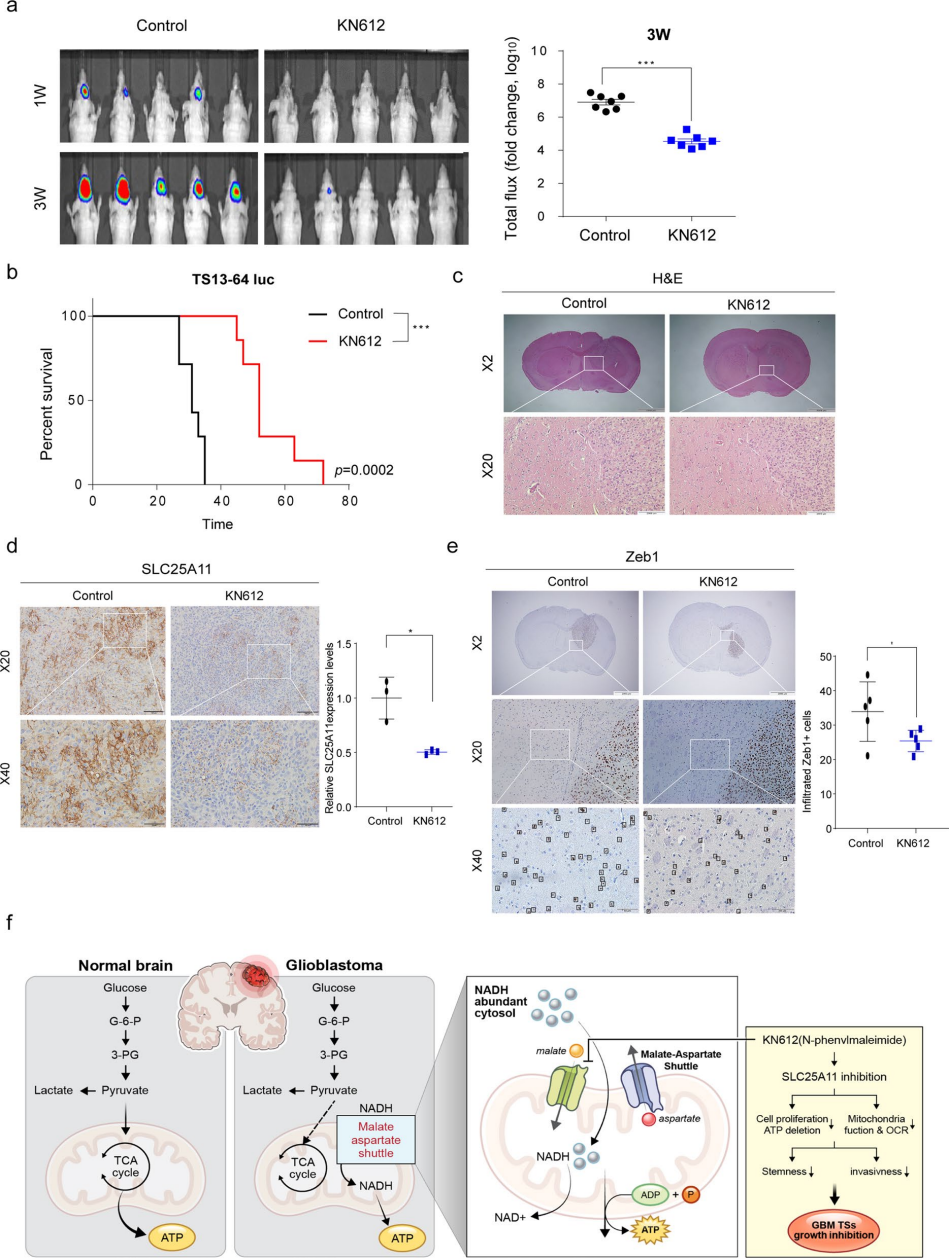
Therapeutic effects of KN612 in an orthotopic xenograft model. TS13-64 luc cells were pretreated with 10 µM of KN612 for 72 h. Control (n = 5), KN612 (n = 5). **a** Tumor volume was measured using bioluminescence imaging. Signal intensity was quantified as total photon flux from tissues. One-way ANOVA with Tukey’s post hoc test (*p < 0.05). **b** Kaplan–Meier curves were used to estimate survival probabilities, and statistical significance was evaluated using the log-rank test. Log-rank test (*p* < 0.05). **c** Hematoxylin and eosin (H&E) staining (magnification × 2, × 20) was performed on brain sections from sacrificed mice to assess the size and extent of tumor masses. **d**, **e** Immunohistochemistry was used to examine SLC25A11 protein expression via brown staining, and to identify invading cells via Zeb1 staining (magnification × 2, × 20, × 40). Zeb1 quantification was conducted using images captured at × 40 magnification. In total, 10 randomly selected images per group were analyzed to quantify the number of infiltrating Zeb1-positive cells (means ± standard error of mean [SEM]; *p < 0.01 compared with control). Hematoxylin (blue) was used to counterstain nuclei in all images. **f** Schematic overview of the study. KN612 inhibits SLC25A11 expression, inducing bioenergetic reprogramming and effectively reducing mitochondrial function, stemness, and invasiveness in GBM cells, ultimately leading to suppressed tumor growth

To confirm the role of KN612 in vivo, we established an orthotopic xenograft model by injecting nude mice with GBM cells pretreated with KN612. Bioluminescence intensity was calculated based on the total flux (photons/s) in each group 3 weeks after injection with KN612 pretreated cells. Tumor size was smaller in the KN612 treatment group than in the control group (Fig. 6a). Kaplan–Meier survival analysis significantly extended the survival duration of the mice (Fig. 6b). Histological examination of brain tissues using H&E indicated that KN612 effectively suppressed the size and extent of tumor masses (Fig. 6c). Immunohistochemical assay showed that KN612 treatment significantly downregulated SLC25A11 expression in the tumors compared with that in the control group (Fig. 6d). Moreover, KN612 treatment reduced the number of Zeb1-positive cells surrounding the primary tumor mass (Fig. 6e). Overall, these findings indicate that KN612 inhibits tumor invasion by suppressing SLC25A11 expression, suggesting that SLC25A11 may plays an important role in promoting tumor growth.

## Discussion

Recent advancements in targeting cancer cell energy metabolism pathways have emerged as a promising therapeutic approach [2, 12–15, 17]. In our previous study, we demonstrated that regulating glycolysis-related processes reduced the proliferation of GBM TSs, while the inhibition of ALDH and OxPhos decreased the stemness, invasion ability, and viability of CSCs [2]. Controlling metabolism and cellular energetics in malignant cells is a promising conceptual pathway for cancer treatment [22]. This study aimed to evaluate the impact of MAS inhibition on the energy metabolism, stemness, and invasiveness of GBM TSs. Specifically, we investigated the ability of KN612, a potent inhibitor of SLC25A11, to block NADH transport, induce bioenergetic disruption, and effectively suppress GBM TS growth. Our findings revealed a significant upregulation of SLC25A11 expression in GBM TSs compared to human normal cells (Fig. 1a, b). This suggests that targeting SLC25A11 may suppress tumor progression in GBM. Indeed, inhibition of SLC25A11 in glioblastoma cells resulted in reduced cell viability, energy depletion, and mitochondrial dysfunction (Fig. 1d–g), highlighting the potential of targeting this gene as a therapeutic strategy for MAS inhibition in GBM treatment.

The anticancer potential of MAS inhibition has been demonstrated in several studies [11, 15, 23, 24]. Lee et al. reported that MAS inhibition suppresses ATP production and mitochondrial activity, thereby inhibiting tumor growth and metastasis [15]. Yoshida et al. and Wang et al. found that MAS inhibition induces cancer cell proliferation arrest, cell cycle disruption, and ATP level reduction [11, 23], while Muthu et al. showed that it enhances drug sensitivity and apoptosis in GLUL-deficient cells [24]. These studies underscore the therapeutic potential of MAS inhibition across various cancer types. Consistently, our study confirmed that KN612-mediated downregulation of SLC25A11 reduced cell viability, ATP production, and NADH levels (Fig. 2d-e). These results further suggest that MAS inhibition could be a viable therapeutic strategy for GBM, while also affecting key phenotypes of GBM TSs, including stemness and invasiveness.

To avoid excessive cytotoxicity while observing metabolic inhibition, we selected a sublethal concentration of 10 μM (20% inhibitory concentration; IC20). Higher concentrations resulted in widespread cell death, making it difficult to analyze specific metabolic alterations [19, 25], whereas non-lethal doses allowed us to evaluate the role of MAS inhibition in GBM TS. At this concentration, KN612 was found to induce mitochondrial dysfunction and apoptosis in GBM TSs (Fig. 2a-c). While this dosage might be insufficient to eliminate all cancer cells, leaving room for survival pathways and resistance development, KN612 effectively induced mitochondrial dysfunction and apoptosis in GBM TSs (Fig. 3). As shown in Fig. 3d, PI-positive necrotic cells (Q1, PI⁺/FITC⁻) in the untreated control group are likely due to inherent characteristics of TS culture systems [20, 21]. In GBM TS cultures, PI-positive cells are observed even in untreated controls, potentially due to factors such as oxidative stress, metabolic heterogeneity, or mitochondrial dysfunction [26–29]. Cancer stem cells (CSCs) are known to contribute to therapeutic resistance and tumor recurrence. Additionally, GBM TS cells exhibit high invasiveness into normal brain parenchyma, resulting in poor patient prognosis [30, 31]. We investigated the effects of ATP depletion and cell death on the key phenotypes of GBM TSs, including stemness and invasiveness. KN612 effectively suppressed stemness and invasiveness in GBM TSs (Fig. 4), with the inhibition of stemness suggesting potential efficacy in KN612 therapies. Furthermore, DEG analysis revealed that KN612 upregulated genes associated with cell cycle arrest (Fig. 5). Additionally, in vivo experiments demonstrated that KN612 effectively suppressed tumor formation and significantly prolonged survival (Fig. 6). These results highlight the robust anticancer effects of KN612 and its clinical potential.

Despite the significant findings, this study has some limitations. The low blood–brain barrier (BBB) permeability of KN612 remains a critical challenge (Supplementary fig. S6a-c). Although preclinical methods were employed in this study, developing drug delivery systems capable of effectively crossing the BBB is essential for GBM treatment. One promising strategy is receptor-mediated transcytosis, which facilitates the transport of target molecules across cells without compromising barrier integrity. Aptamer-based delivery systems could enhance BBB penetration while maintaining its integrity [32]. Aptamers could serve as delivery vehicles for KN612, potentially overcoming the current challenges.

While MAS inhibition by KN612 effectively suppressed GBM TS growth, the metabolic adaptability of GBM cells presents a major obstacle to achieving sustained therapeutic efficacy. One key adaptive mechanism involves metabolic reprogramming toward fatty acid oxidation (FAO) under metabolic stress conditions such as glucose deprivation or MAS inhibition [33, 34]. Also, the ketogenic diet (KD), which promotes a metabolic shift from glycolysis to ketone body utilization, could be proposed as a complementary strategy for GBM treatment [35–37]. We evaluated the combinatorial effects of KN612 with metabolic modulators targeting FAO and ketogenesis pathways. However, no synergistic effects were observed with etomoxir (a CPT1 inhibitor) or 5-hydroxybutyrate (5-HB) in TS13-64 cells (Supplementary fig. S7a-b). These findings suggest that GBM cells may utilize alternative metabolic pathways beyond FAO and ketogenesis to compensate for MAS inhibition.

Future studies should explore strategies combining KN612 with standard therapies (e.g., TMZ) or inhibitors targeting alternative metabolic pathways. Additionally, developing novel drug delivery systems to enhance the in vivo efficacy of KN612 is crucial. Despite these limitations, our findings provide strong evidence for the potential of MAS inhibition as a novel metabolic strategy for GBM treatment, highlighting the need for future investigations into optimized delivery systems and combination strategies.

## Conclusions

KN612 possesses potential application in GBM treatment and can be combined with currently available treatments. In addition to conventional therapies, this innovative approach may enhance cancer management and extend the lifespan of patients with cancer.

## Supplementary Information

Supplementary Material 1.

## Data Availability

No datasets were generated or analysed during the current study.

## Abbreviations

ALDH: Aldehyde dehydrogenase
GBM: Glioblastoma
GOT: Glutamic-oxaloacetic transaminase
MAS: Malate-aspartate shuttle
MDH: Malate dehydrogenase
NHA: Normal human astrocytes
OCR: Oxygen consumption rate
OxPhos: Oxidative phosphorylation
TSs: Tumorspheres
