# Supplementary material for: N-phenylmaleimide induces bioenergetic switch and suppresses tumor growth in glioblastoma tumorspheres by inhibiting SLC25A11
Source: Cancer Cell Int. 2025 May 22;25:184. doi: 10.1186/s12935-025-03813-y (PMC12096590; doi:10.1186/s12935-025-03813-y)
Supplement: Supplementary file 1 — Supplementary Material 1. [file 12935_2025_3813_MOESM1_ESM.docx]

**Supplementary table 1.** Histopathology of tissue and tumorsphere derived from GBM patients.

| **Sample type** | **Pathology** | **Molecular markers** | | **Subtype** | | |
| --- | --- | --- | --- | --- | --- | --- |
|  |  | ***MGMT***  **promoter** | **Codeletion of**  **1p/19q** | **Verhaak’s [1]** | | **Prognostic [2]** |
| Tissue 13-64 | Glioblastoma,  *IDH*_wildtype | Unmethylated | Intact/Intact | Classical | Invasive | |
| TS13-64 | Glioblastoma,  *IDH*_wildtype | Unmethylated | Intact/Intact | Classical | Intermediate | |
| Tissue 15-88 | Glioblastoma,  *IDH*_wildtype | Unmethylated | Intact/Intact | Mesenchymal | Invasive | |
| TS15-88 | Glioblastoma,  *IDH*_wildtype | Unmethylated | Intact/Intact | Proneural | Intermediate | |
| Tissue 13-30 | Glioblastoma,  *IDH*_wildtype | Unmethylated | Intact/Intact | Mesenchymal | Invasive | |
| TS13-30 | Glioblastoma,  *IDH*_wildtype | Unmethylated | Intact/Intact | Mesenchymal | Invasive | |

Presence of IDH mutations, methylation status of the O6-DNA MGMT promoter,

presence of co-deletion of chromosome 1p/19q, Verhaak’s subtype, and prognostic subtype of tissue are summarized.

**References**

1. Verhaak RGW, Hoadley KA, Purdom E, et al. Integrated Genomic Analysis Identifies Clinically Relevant Subtypes of Glioblastoma Characterized by Abnormalities in PDGFRA, IDH1, EGFR, and NF1. *Cancer Cell*. 2010; 17(1):98-110.
2. Park J, Shim JK, Yoon SJ, Kim SH, Chang JH, Kang SG. Transcriptome profiling-based identification of prognostic subtypes and multi-omics signatures of glioblastoma. *Scientific Reports*. 2019; 9.

**Supplementary table 2.** Antibodies used in this study

| **Antibody** | **Manufacture** | **Cat.Number** | | **Dilution** |
| --- | --- | --- | --- | --- |
| Sox2 | Merck Millipore | AB5603 | 1:1000 | |
| MDH1 | Novus Biologicals | NBP1-89515 | 1:1000 | |
| MDH2 | Novus Biologicals | NBP1-32259 | 1:1000 | |
| Nestin | Novus Biologicals | NB100-1604 | 1:1000 | |
| CD133 | Cell Signaling Technology | 64326T | 1:1000 | |
| PDPN | Cell Signaling Technology | 9047T | 1:1000 | |
| Zeb1 | Cell Signaling Technology | 3396T | 1:1000 | |
| Snail | Cell Signaling Technology | 3879T | 1:1000 | |
| N-cadherin | R&D Systems | MAB13881 | 1:1000 | |
| SLC25A11 | Santa Cruz Biotechnology | sc-515593 | 1:2000 | |
| Oct3/4 | Santa Cruz Biotechnology | sc-101534 | 1:2000 | |
| Twist | Santa Cruz Biotechnology | sc-81417 | 1:2000 | |
| GAPDH | Santa Cruz Biotechnology | sc-32233 | 1:2000 | |


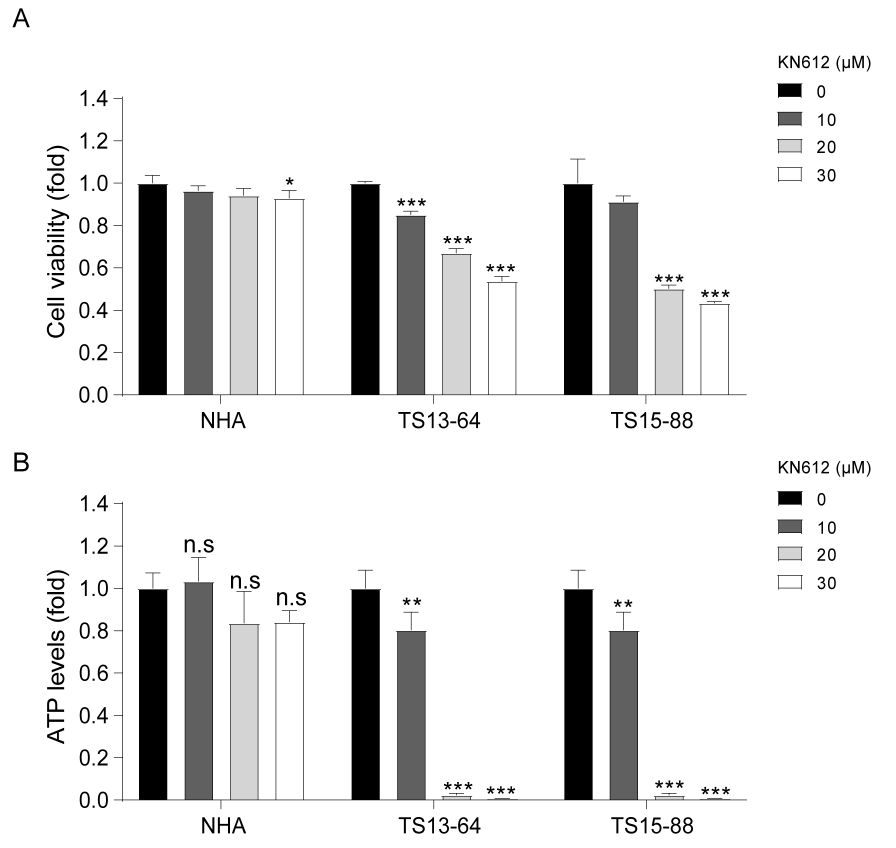


**Supplementary fig. S1.** Biological Effects of KN612 on NHA Cell and GBM TSs. The effects of KN612 treatment on cell viability and ATP levels in NHA and GBM TSs were evaluated. **a)** MTT assay was performed to assess the effects of KN612 treatment for 72 h on the viabilities of NHA and GBM TSs. **b)** ATP levels in NHA and GBM TSs were measured using a luciferase assay after treatment with various concentrations of KN612 for 72 h, and the same trends were noted. Differences between groups were evaluated using Welch’s ANOVA and Games-Howell post hoc tests. The data are presented as the mean ± standard deviation (SD); **p* < 0.05, ***p* < 0.01 and ****p* < 0.001.


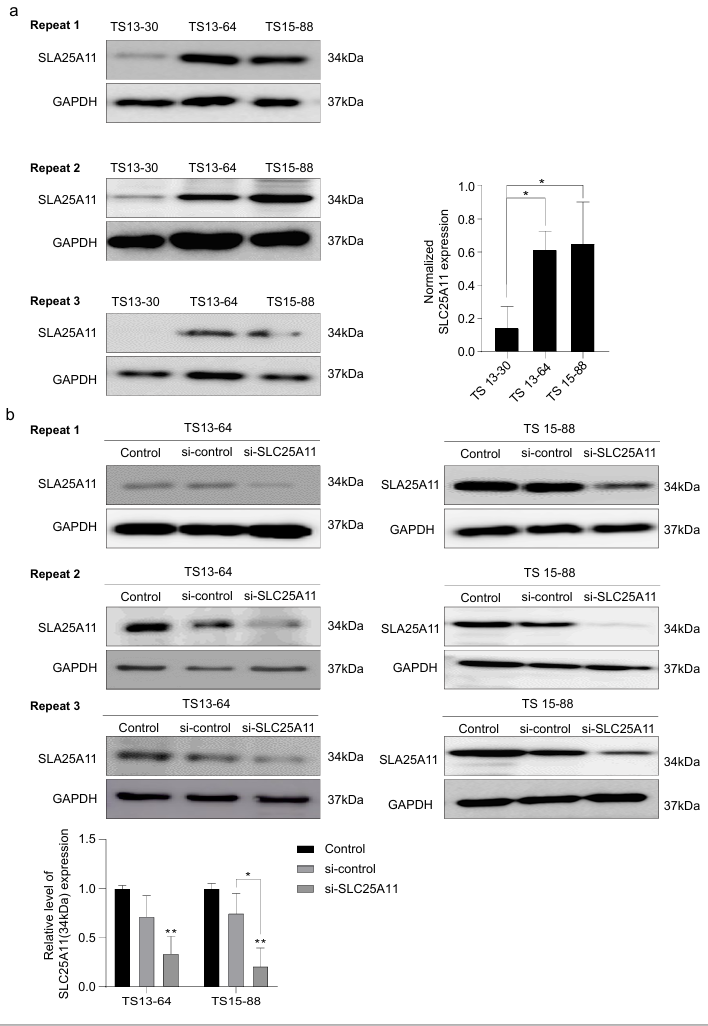


**Supplementary fig S2.** Protein expression quantification and densitometry in fig.1. In GBM TS, the expression level of SLC25A11 was assessed, and cells with high gene expression, TS13-64 and TS15-88, were selected. siRNA treatment was applied for 48 h to induce SLC25A11 knockdown, followed by measurement and analysis. **a)** Images of results from three independent experimental repeats and densitometry analysis representing the quantification of protein expression in Fig. 1b. **b)** Images of results from three independent experimental repeats and densitometry analysis representing the quantification of protein expression in Fig. 1c. The expression levels of proteins in each group were compared using Welch’s ANOVA and Games-Howell post hoc tests to assess statistical significance (means ± SD; **p* < 0.05, and ***p* < 0.01).


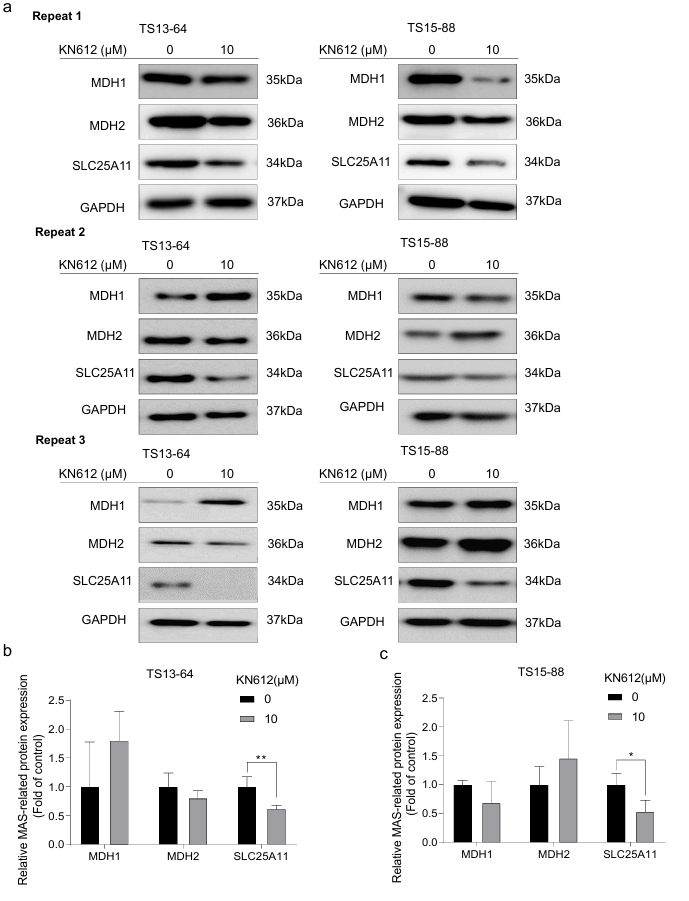


Supplementary fig S3. Protein expression quantification and densitometry in figure 2b. MDH1, MDH2, and SLC25A11 protein levels were assessed 72 h following KN612 treatment. a) Images of results from three independent experiments and densitometry analysis representing the quantification of protein expression in Fig. 2b. b,c) The expression levels of MAS-related proteins, MDH1, MDH2, and SLC25A11, in each group were compared using an unpaired Student's t-test to assess statistical significance in the TS13-64 and TS15-88.


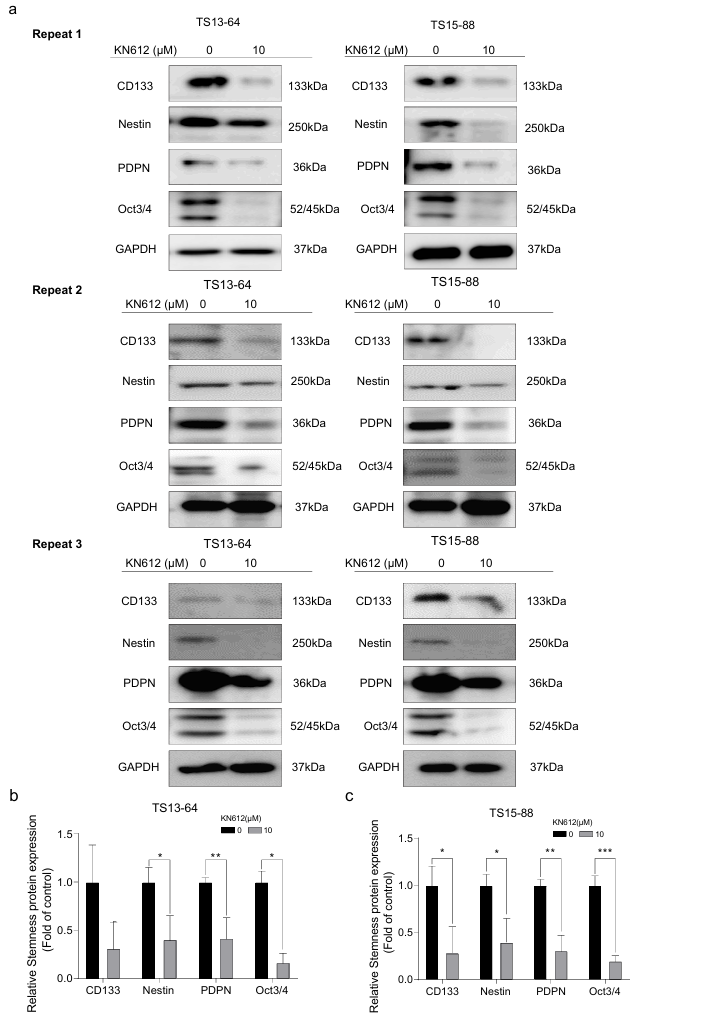


Supplementary fig. S4. Protein expression quantification and densitometry in fig. 4c. Protein levels of stemness-related proteins were assessed 72 h following KN612 treatment. a) Show images of results from three independent experiments and densitometry analysis representing the quantification of protein expression in Fig. 4c. b,c) The expression levels of the stemness-related proteins, CD133, Nestin, PDPN, Oct3/4 in each group were compared using an unpaired Student's t-test to assess statistical significance in TS13-64 and TS15-88.


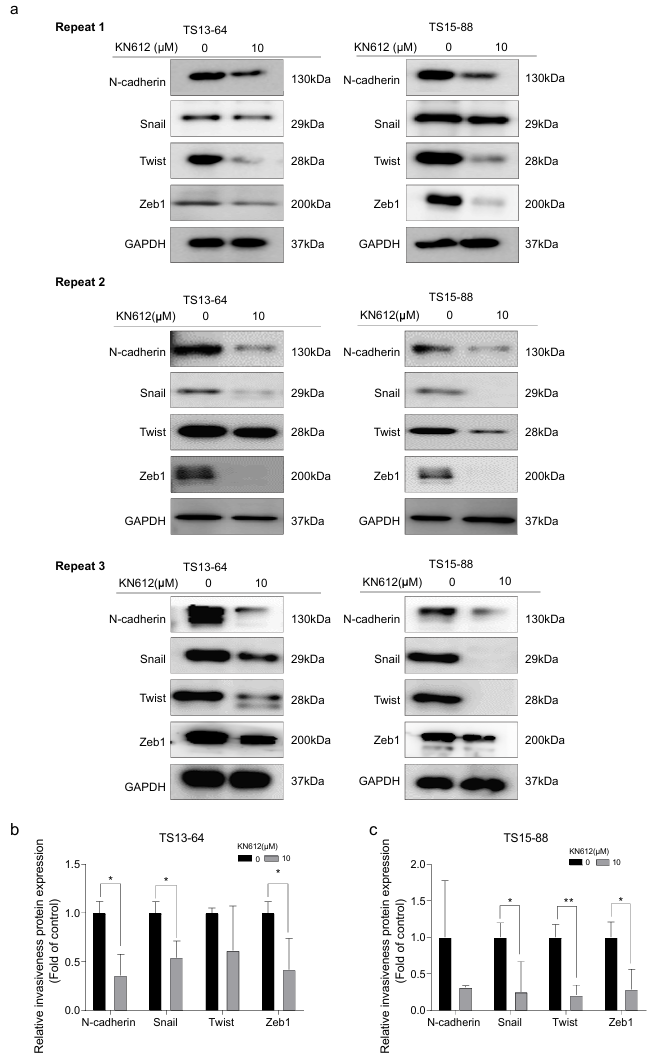


Supplementary fig. S5. Protein expression quantification and densitometry in fig.4d. The protein levels of invasiveness-related proteins were assessed 72 h following KN612 treatment. a) Images of results from three independent experiments and densitometry analysis representing the quantification of protein expression in Fig.4d. b,c) The expression levels of the invasiveness-related proteins, N-cadherin, Snail, Twist and Zeb1 in each group were compared using an unpaired Student's t-test to assess statistical significance in TS13-64 and TS15-88.

**
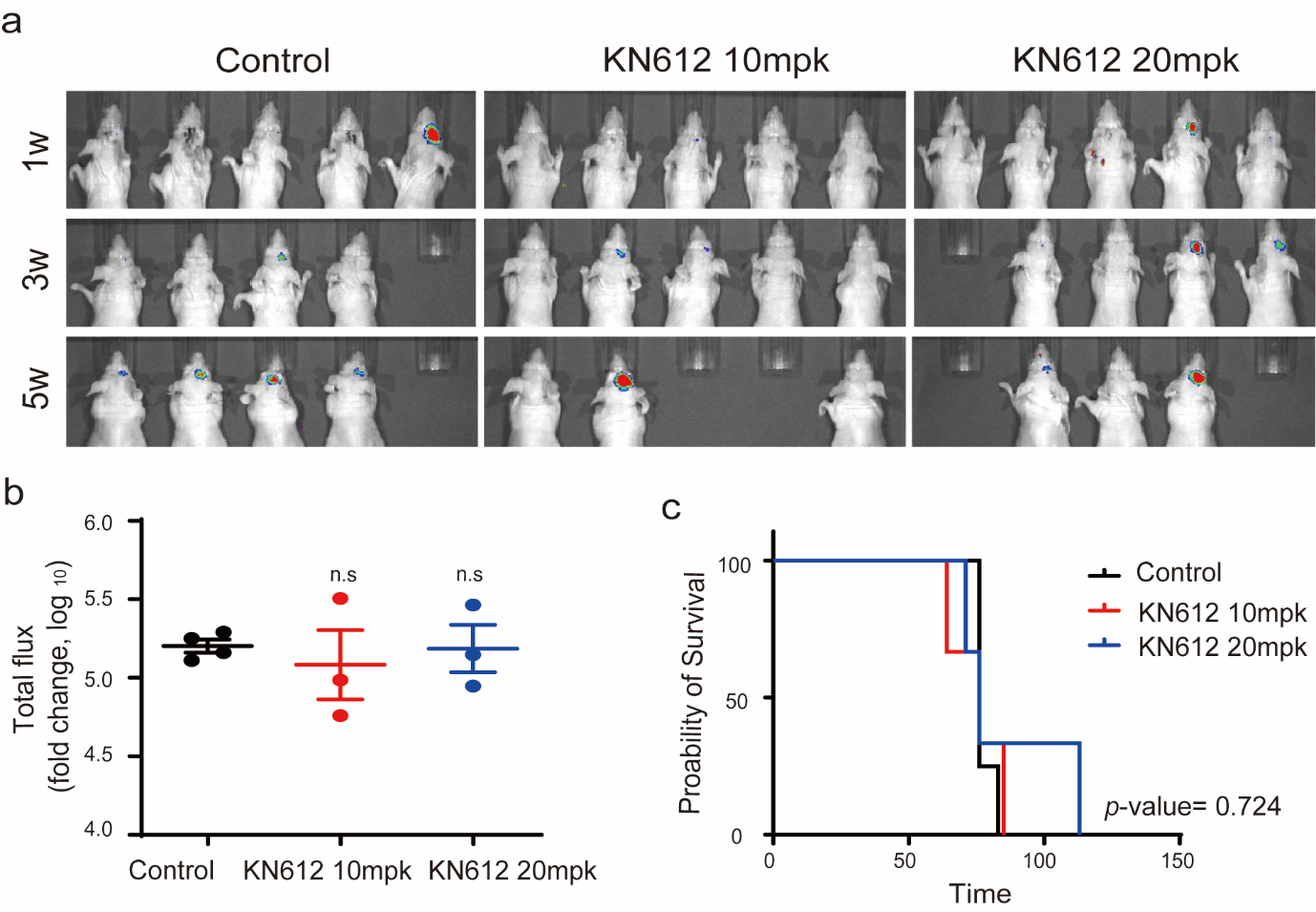
**

**Supplementary fig. S6.** Therapeutic effects of KN612 in orthotopic xenograft models. TS13-64-luc cells were implanted into the right frontal lobe of mice using a guide-screw system. KN612 was administered orally daily for 4 weeks (5% DMSO, 10% cremophor, 85% PBS). **a)** Tumor volumes were monitored using bioluminescence imaging, and total flux from the region of interest (ROI) in the mouse brain was measured using an In Vivo Imaging System (IVIS). **b)** Quantification of total flux in each group. Statistical analysis was performed using one-way ANOVA followed by Tukey’s post hoc test (n.s: not significant). **c)** Kaplan–Meier survival curves for each group, with comparisons conducted using the log-rank test (*p*-value= 0.724).


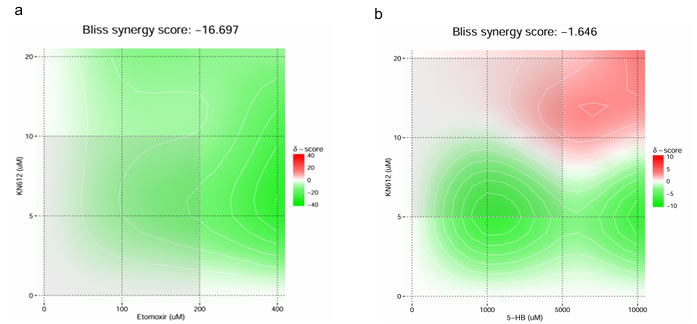


**Supplementary fig. S7.** Combined effects of KN612 and drugs targeting fatty acid oxidation and ketogenesis in TS13-64 cells. The cells were treated with combinations of KN612 and 5-hydroxybutyric acid (5-HB) or Etomoxir for 72 h, followed by Bliss synergy score calculation. **a)** The Bliss synergy score for the combined treatment of Etomoxir and KN612, indicating no synergistic effect in TS13-64 cells (-16.697 points). **b)** The Bliss synergy score for the combined treatment of 5-HB and KN612, showing no synergistic effect in TS13-64 cells (-1.646 points).
